# Randomized, double-blind, placebo-controlled study of the analgesic effect of intraoperative esmolol for laparoscopic gastroplasty^1^

**DOI:** 10.1590/s0102-865020200040000008

**Published:** 2020-06-12

**Authors:** Vinicius Barros Duarte de Morais, Rioko Kimiko Sakata, Ana Paula Santana Huang, Leonardo Henrique da Cunha Ferraro

**Affiliations:** I MD, Pain Sector, Department of Surgery, Universidade Federal de São Paulo (UNIFESP), Brazil, Design of the study, acquisition of data, final approval.; II PhD, Pain Sector, Department of Surgery, UNIFESP, Sao Paulo-SP, Brazil, Design of the study, manuscript writing, critical revision, final approval.; III Master, Pain Sector, Department of Surgery, UNIFESP, Sao Paulo-SP, Brazil. Acquisition of data, final approval.; IV PhD, Pain Sector, Department of Surgery, UNIFESP, Sao Paulo-SP, Brazil. Manuscript writing, final approval.

**Keywords:** Esmolol, Analgesia, Intraoperative Period, Gastroplasty

## Abstract

**Purpose:**

To evaluate the analgesic effect of esmolol in patients submitted to laparoscopic gastroplasty.

**Methods:**

Forty patients aged between 18 and 50 years with American Society of Anesthesiologists (ASA) physical status scores of II and III who underwent gastric bypass were allocated to two groups. Group 1 patients received a 0.5-mg/kg bolus of esmolol in 30 mL of saline before induction of anesthesia, followed by an infusion at 15 µg/kg/min until the end of surgery. Group 2 patients received 30 mL of saline as a bolus and then an infusion of saline. Anesthesia included fentanyl (3 µg/kg), propofol (2-4 mg/kg), rocuronium (0.6 mg/kg), and 2% sevoflurane, with remifentanil if necessary. The following parameters were evaluated: pain intensity over 24h, remifentanil consumption, the first analgesic request, morphine consumption, and side effects.

**Results:**

Pain intensity was lower in the esmolol group except at T0 (after extubation) and 12h postoperatively. Remifentanil supplementation, recovery time, and postoperative morphine supplementation were lower in the esmolol group. No differences in the time to the first analgesic request or side effects were found between the groups.

**Conclusion:**

Intraoperative esmolol promotes reductions in pain intensity and the need for analgesic supplementation without adverse effects, thus representing an effective drug for multimodal analgesia in gastroplasty.

## Introduction

Postoperative analgesia and recovery of patients undergoing bariatric surgery are challenging. Opioids are effective in relieving postoperative pain; however, especially in morbidly obese, these drugs are associated to side effects^1,2^. Other drugs are often given to increase the analgesic effect of opioids and decrease the incidence and severity of side effects. Also, lower half-life drugs are recommended for these patients^1^. Thus, multimodal analgesia with drugs of different actions is the most prudent approach for morbidly obese patients. A combination of short-acting drugs with a focus on opioid reduction can reduce vomiting and pulmonary complications, enabling early ambulation and shortening the hospital stay^3^.

Beta-adrenergic antagonists, such as esmolol, have been used in some studies for postoperative multimodal analgesia^3,4^. Several mechanisms have been proposed for the analgesic action of beta-blockers such as: modulation of calcium and potassium channels and adrenergic activity, inhibition of sodium channels and facilitation of inhibitory neurotransmitter release^3,5,6^. Esmolol may have analgesic effects, blocking tetrodotoxin-resistant sodium channel activity as lidocaine, in dorsal root ganglion neurons^7^. Facilitation of inhibitory transmitter release, through a mechanism involving Ca2+-entry but in a β1-adrenoceptor-independent manner is another mechanism of the antinociceptive effect of esmolol^5^.

The analgesic effect of esmolol is controversial. In some studies, there was a reduction in postoperative opioid consumption^3,8,9^ and pain intensity^10^; however, in other study, esmolol failed to promote an analgesic effect^11^.

The primary objective of this study was to evaluate the effect of esmolol infusion on pain intensity after gastroplasty. The secondary objective was to assess the remifentanil and morphine consumption, the time to require analgesic, and the incidence of side effects. The hypothesis of the study is that esmolol promotes a decrease in pain intensity and in the total opioid consumption.

## Methods

The study was prospective, randomized, comparative, double-blind, and paired sample. The sample size was calculated using the SPSS17^®^ software. The test of choice was Student’s t for two independent paired sample with 80% for power and alpha at 5%. To calculate the sample, it was set 2.4 point for the pain score for the difference between the groups. The result was 19 participants in each group, and it was allocated 20 in each group.

The study was registered at the Brazilian Clinical Trials Registry (ReBec-9w3k77). The data were collected at IGESP Hospital, São Paulo, Brazil.

After approval from the Ethics Committee (CAAE N^o^ 83115117.5.0000.5450) and signing the Consent Form, 40 patients with 18 to 50 years old, of both genders, physical status II or III by American Society of Anesthesiology (ASA), submitted to laparoscopic bypass gastroplasty_,_ were included in the study. This study was conducted in accordance with the Declaration of Helsinki. Patients with drug allergy; respiratory, renal, hepatic, cardiovascular or psychiatric disease; cognitive alteration; use of beta-adrenergic antagonists; or use of illicit drugs, were excluded.

Participants were randomly drawn and allocated to one of the groups. The randomization was performed by the Randomizer® program. The drawings for allocation in the groups were made by numbers placed in an envelope. On the day of surgery before the onset of anesthesia, the pharmacist opened the participant’s envelope and prepared the solution according to the draw, with esmolol or saline solution. The anesthesiologist and the evaluator did not know which group the participant belonged to until the end of the study. In case of an emergency, the anesthesiologist caring for the patient could break the protocol and see the group assignment.

Participants were allocated into two groups. Group 1 (Esmolol) patients received a 0.5mg/kg bolus of esmolol in 30 mL of saline before induction of anesthesia, followed by infusion of 15 µg/kg/min until end of surgery; group 2 (Control) patients received a 30mL bolus of saline and infusion of saline in the same volume as G1.

Monitoring was performed with a cardioscope, capnograph, pulse oximeter, noninvasive blood pressure, and neuromuscular blockade device.

Before induction of anesthesia, both groups received 2g of dipyrone and 40mg of parecoxib. Anesthesia was performed with fentanyl (3ug/kg; by real bodyweight), propofol (2-4mg/kg) and rocuronium (0.6mg/kg and as required), 50% oxygen and 2% sevoflurane. Neuromuscular block was maintained by train of four (TOF), and a post-tetanus count of II, during the surgery. Remifentanil intraoperatively (0.05 to 0.2ug/kg/min) was given if the heart rate was greater than 15% and the systolic blood pressure was greater than 20% of the baseline values. The baseline heart rate and systolic blood pressure was defined as the mean of the two lowest measurements recorded during the 3- to 5-minute interval prior to anesthetic induction. In case of hypotension, defined as systolic blood pressure less than 80 mmHg or mean arterial pressure lower than 60 mmHg, a bolus of ephedrine (0.5mg IV) was administered; in case of bradycardia, defined as heart rate <50, a bolus of atropine was administered (0.5mg). Intra-abdominal pressure by pneumoperitoneum was the same (15mmHg) for all patients. The surgical technique was laparoscopic gastric bypass.

Before extubation, 2mg/Kg of sugammadex was administered; with an additional dose (2 mg/kg), if necessary, until it reached >90% TOF. Patients were kept in the recovery unit and received oxygen (5 L/min), until saturation was greater than 92% in ambient air for 10 min. Side effects and complications were noted. Patients with heart rate < 45bpm or mean blood pressure <60 mmHg were withdrawn from the study and treated.

Postoperative pain was treated with intravenous morphine (5mg per dose) as required. The following were evaluated: consumption of intra-operative remifentanil; time to first drug requirement; intensity of pain by numerical scale from 0 to 10 after extubation (T0) and 30 minutes,1h, 2h, 6h, 12h and 24h; morphine dose in 24h; and side effects

The primary outcome was pain intensity reduction. The secondary outcomes were remifentanil requirement, morphine dose, and adverse effects. No change in outcome was made after the trial commenced.

The results were submitted to statistical analysis by SPSS^®^ program. Sample normality test was performed using Shapiro –Wilk test. The following tests were used: Wilcoxon for age, weight, height, body mass index, duration of surgery, amount of remifentanil, time to recovery, time to first supplementation of morphine, dose of morphine in 24h, and pain intensity; chi-square test for number of participants requiring remifentanil and morphine supplementation; and Fisher’s test for side effects. The level of statistical significance was p<0.05.

## Results

**Figure 1:**
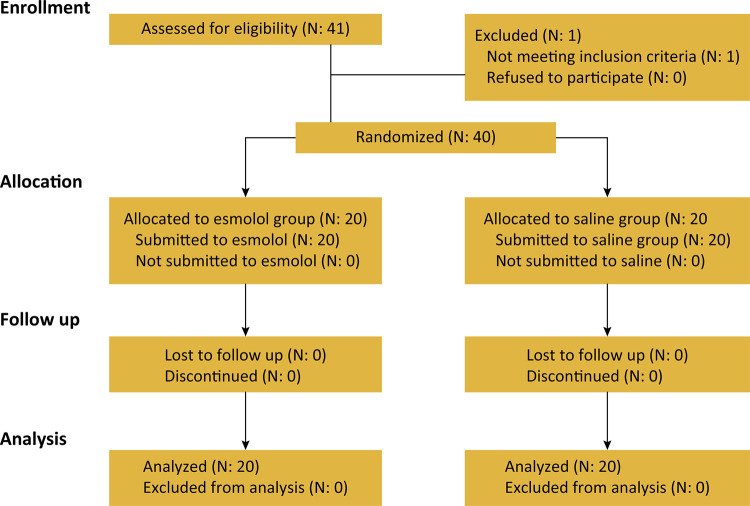
CONSORT flowchart.

The CONSORT flowchart is shown in Figure 1. There was no difference in demographic data, duration of surgery and ASA between the groups (Table 1).

**Table 1:** Characteristics of participants according to age, height, weight, body mass index, duration of surgery (mean± SD); gender and ASA physical status (number).

	Esmolol	Saline	P
Age (years)	35.8±10.9	33.2±8.7	0.379
Gender: M / F	3 / 17	3 / 17	NC
Weight (kg)	105.6±20.2	109.8±11.2	0.148^:^
Height (cm)	161.9±8.0	164.4±9.7	0.456
BMI (kg.m^-2^)	40.1±5.5	40.7±3.3	0.148^:^
ASA: I / II	0 / 20	0 / 20	NC
Duration of surgery	104.3±14.3	112.8±12.5	0.078

Wilcoxon test; BMI: body mass index; ASA: American Society of Anesthetists; NC: not calculated

Pain intensity was lower over 24 h in the esmolol group, except at T0 and after 12 h (Table 2). There was a need for supplementation with postoperative morphine in 17 patients from the esmolol group and 20 from the saline group; the morphine dose over 24 h was lower in the esmolol group. There was no difference in time to the first supplementation (Table 3). Intraoperative remifentanil supplementation was required in 3 patients in the esmolol group and in 17 in the saline group, and the dose was higher in the saline group. The time to wake up was shorter in the esmolol group. There were no differences in side effects between groups (Table 4).

**Table 2:** Intraoperative supplementation with remifentanil (dose and number of patients needing supplementation), time until recovery, time until first postoperative first supplementation, and morphine (dose in 24 h and number of patients needing supplementation) (mean ± SD).

	Esmolol	Saline	P
Intraoperative remifentanil (µg)	620.0±182.5	1058.8	0.001^†^:
Number who needed remifentanil	3	17	0.001^‡^
Time for recovery (min)	9.0 ± 3.4	12.5 ± 3.7	0.006^†^:
First supplementation (min)	37.1±16.9	33.0±19,2	0.535^†^:
Dose of morphine (mg)	7.0 ± 4.4	13.0 ± 5.7	0.002^†^:
Number who used morphine	17	20	0.100^‡^

^†^: Wilcoxon test; ^‡^: qui-square; SD: standard deviation

**Table 3:** Intensity of pain at recovery (T0), after 30 minutes, 1, 2, 6, 12 and 24 h, according to numerical scale – median (minimum- maximum).

	Esmolol	Saline	P
T0	0 (0 - 8)	0 (0 - 8)	0.180
30 min	5 (0 - 8)	6 (2 - 10)	0.032
1 h	5 (0 - 9)	8 (3 - 10)	0.004
2 h	2 (0 - 6)	5 (2 - 8)	0.002
6 h	3 (0 - 5)	4 (1 - 7)	0.047
12 h	1 (0 - 2)	1 (0 - 3)	0.262
24 h	0 (0 - 2)	1 (0 - 2)	0.029

Wilcoxon test; T0= at recovery of consciousness

**Table 4:** Side effects – number (%).

	Esmolol	Saline	P
Nausea	6 (30%)	9 (45%)	0.515
Vomiting	0 (0%)	0 (0%)	NC
Somnolence	9 (45%)	7 (35%)	1.000
Hypotension	3 (15%)	3 (15%)	1.000
Bradycardia	2 (10%)	2 (10%)	1.000
Bronchospasm	0 (0%)	1 (5%)	1.000

Fisher test; NC: not calculated

## Discussion

We found that intraoperative continuous infusion of esmolol reduce pain intensity in the first 24 h, the morphine dose over 24 h and the amount of remifentanil use during bypass laparoscopic gastroplasty. These results indicated that esmolol may be used effectively to achieve an opioid-sparing effect during surgery and qualitatively better recovery from anesthesia.

Inadequate control of postoperative pain is a common cause of prolonged hospital stay^12^. In this study, analgesia was improved with esmolol, both during surgery and in the postoperative period over 24 hours. This result is similar to literature on other types of surgeries and patients with BMI within the normal range^8,9,13^.

In one study, with a similar dose of esmolol as in this study, it was observed reduction of alfentanil infused during laparoscopic cholecystectomy, and also the dose of tramadol and diclofenac for postoperative pain control^14^.

In hysterectomy, esmolol infusion in non-obese patients, it was a reduced dose of remifentanil, and the postoperative analgesic consumption was lower in the esmolol group. Like our study the nausea and vomit are the same in each group^15^.

Intravenous infusion of esmolol reduced the intraoperative and postoperative analgesic consumption, reduced visual analogue scale scores in the early postoperative period and prolonged the time to first analgesia, in patients undergoing septorhinoplasty^10^.

Remifentanil was used for intraoperative supplementation because although it is liposoluble, its degradation is rapid, and does not enter the lipophilic compartment. It is used safely in obese patients, with the volume of distribution and clearance being similar to that of the non-obese population^16^. Reduction in remifentanil consumption in the esmolol group may have led to lower pain scores because this opioid may cause hyperalgesia^17^.

Intraoperative esmolol may reduce the consumption of opioids and their side effects, such as nausea, vomiting and ileus, with less time to hospital discharge. In this study, a 0.5 mg/kg bolus was given, similar to the majority of literature reports^3,10,15^. The infusion was performed at lower doses than those in the literature for nonobese which include reports of 5µg/kg/min ^10^, 10µg/kg/min ^8^, 30µg/kg/min ^15^, 50ug/kg/min^3,18^. There are no pharmacokinetic models to morbidly obese population, so in our study we use a low dose of fentanyl.

Esmolol was hemodynamically safe, and there were no differences in the incidence of bradycardia or hypotension between the groups, as in the studies of the literature^8,9-11,15,18^. In this study, esmolol was not associated with significant bradycardia. Hypotension was related to boluses greater than 0.5mg in one review^19^. In one study in the esmolol group, the heart rate was slightly higher than that in the placebo group ^10^. In another study, more bradycardia was observed with esmolol but without hemodynamic instability^19^. Other studies have demonstrated hemodynamic benefits for orotracheal intubation^20^ and reduced myocardial oxygen consumption by preventing adverse events during surgery^21^.

There was no significant difference in the incidence of nausea and vomiting, unlike in previous studies^14,22^, but nausea was less common in the esmolol group. However, a preventive antiemetic drug was administered in this study for both groups. Prevention of postoperative nausea and vomiting is very important, especially after gastroplasty. Due to the high incidence of nausea and vomiting in this type of surgery it is not possible to keep them without prophylactic antiemetic. There was one case of bronchospasm, but with no correlation with esmolol, as it was observed in the saline group.

## Limitations

Reduced incidence of nausea and vomiting by the administration of prophylactic antiemetic may have influenced the absence of difference in these adverse effects.

## Conclusion

Intraoperative esmolol promotes reduction in pain intensity, and need for supplementation, without increased risks and represents an effective drug for multimodal analgesia in obese patients submitted to gastroplasty.

## Footnotes

1 Research performed at Instituto de Gastroenterologia, Sao Paulo-SP, Brazil. Part of Master degree thesis, Interdisciplinary Surgical Science Postgraduate Program, Department of Surgery, UNIFESP. Tutor: Rioko Kimiko Sakata.
